# The effects of aging, sex, and tumor burden on the peripheral blood immune cell profile and absolute counts

**DOI:** 10.7150/jca.93542

**Published:** 2024-05-28

**Authors:** Zenghui Xu, Donglei Han, Yan Zhang, Li He, Ou Tong, Zhongyang Han, Zhenlong Ye, Kexin Xiang, Yan Sun, Qijun Qian

**Affiliations:** 1 Shanghai University Mengchao Cancer Hospital, Shanghai, China.; 2 Shanghai Cell Therapy Research Institute, Shanghai, China.; 3 Shanghai Cell Therapy Group Co., Ltd, Shanghai, China.; 4 Henan Cell Therapy Group Co., Ltd, Zhengzhou, China.; 5 School of Medicine, Shanghai University, Shanghai, China.

**Keywords:** peripheral immune status, aging, sex, tumor metastasis

## Abstract

**Background:** To better assess the peripheral immune status and aid in the early diagnosis and prognosis of tumors, we compared the proportion and absolute counting of peripheral immune cell subsets in healthy individuals and tumor patients of varying ages, taking into account the impact of sex and tumor metastasis.

**Methods:** We used peripheral blood mononuclear cell (PBMC) samples from 520 patients with various tumor types and 109 healthy volunteers. The absolute numbers of lymphocytes and monocytes were identified by an automated blood analyzer, and multi-parameter flow cytometry was used to examine the subsets of natural killer (NK) cells (CD3-CD16+CD56+), T cells (CD3+CD4+/CD8+), and mononuclear cells (CD14+) in PBMC.

**Results:** The percentage of T cells (CD3+) in peripheral blood mononuclear cells (PBMC) was 55.83% VS 45.54% (P<0.0001) between healthy volunteers and tumor patients, showing a significant downward trend. Meanwhile, the percentages of monocytes (CD14+) and NK cells (CD3-CD16+CD56+) showed a significant upward trend. Single factor or multifactor analysis yielded identical findings on the proportion of PBMC between healthy individuals and patients with different malignancies, considering the three confounding variables of age, sex, and tumor metastasis.

**Conclusion:** The proportion and absolute counting of acquired immune T cells, innate immune NK cells, and monocytes in PBMCs all exhibit substantial changes between cancer patients and healthy individuals, and the differences are influenced by age, sex, and tumor progression.

## Introduction

While several hypotheses have been proposed to explain tumor development, the most popular one is that tumors arise when a series of gene mutations builds up over time. Human genome sequencing and studies of tumor genomes have improved our understanding of the roles played by driver and passenger genes in carcinogenesis, paving the way for the creation of a wide variety of medications designed to treat cancer. Surgery, radiation, chemotherapy, and targeted medications have all played key roles throughout the history of tumor treatment, yet none of them has been able to properly treat tumors. The human body, and the immune system, are severely harmed by these therapy approaches 1.

The use of bacterial vaccinations in the treatment of cancer dates to the late 19^th^ century. The basic idea is to utilize infectious microorganisms to stimulate the immune system and kill cancer cells 2. Different adoptive immunotherapies have shown exceptional clinical success and significant commercial growth has been noted since the articles "The era of tumor immunotherapy has come" were published by Nature and JCO in 2011 3, 4. Twenty-two immune checkpoint inhibitors, one TCR-T cell product, and eight CAR T cell products are only some of the 228 tumor treatment-related medications that have been authorized for commercialization across the globe 5, 6. Therefore, immunotherapy is increasingly being regarded as the "fourth pillar" of cancer care.

Immunotherapy represents a breakthrough cancer treatment, but not all patients can ultimately benefit from its administration. The interaction between the host immune system and the tumor microenvironment is critical in determining how well patients may respond to immunotherapy. Phenotype analysis and absolute counting of PBMCs have been proven in studies to predict the immune system status of tumor patients and might be utilized as predictive criteria 7-10. T lymphocytes are the most important effector cells in cellular immunity. They may create cytokines and mediate inflammatory events during the immunological response. In CAR-T immunotherapy, the proportion and count of CD4+ and CD8+ T cells are intensively linked to a curative effect. Natural killer (NK) cells, as a subset of innate lymphoid cells, may prevent primary tumor cell migration to distant organs and tumor cell growth by natural cytotoxicity. In tumor patients, activating receptors of circulating NK cells were diminished, and the capacity to identify and destroy mutant cells was impaired 11, 12. Monocytes provide antitumoral immunity in a variety of ways, including tumoricidal mediator release, lymphocyte recruitment, and differentiation into tumor-associated macrophages and dendritic cells.

Research has shown that the activity of immune cells declines with age, increasing the risk of infection and cancer 13, 14. The key sign of immunological senescence is a decline in the numbers and diversity of T cells in PBMCs 15, 16. Furthermore, the proportions of peripheral immune cells fluctuate across sex. Evidence indicates that the severity of immune system illnesses is strongly related to sex 17. Additionally, it has been discovered that the greater the tumor's malignancy, such as distant metastasis, the stronger the inhibition of immune function and the simpler the tumor's immunological escape 18. Few studies have taken the critical elements of aging, sex, and tumor metastasis into thorough account and conducted synthetical analysis in relevant investigations of the difference in the proportions of peripheral immune cells. Furthermore, there has been no complete exploration of numerous tumor types among adequate sample sizes, leading to contradictions in the findings of different research investigations 18-20.

In this study, we analyzed the impact of aging, sex, and tumor metastasis on the peripheral blood immune system of tumor patients and healthy individuals based on single-factor and multifactorial analyses, large sample size, and multiple tumor types, to accurately evaluate the influence of the immune system in this regard. Accurately assessing the variables influencing patients' immune state and the trend of immune status changes is critical for screening tumor liquid biopsy biomarkers and predicting the associated therapeutic impact.

## Materials and Methods

### Patient selection and study design

Between May 2020 and November 2022, a total of 520 patients with tumors participated in this study at Shanghai Mengchao Cancer Hospital. Eligibility for inclusion was contingent upon the availability of PBMC samples. These samples were obtained before the commencement of surgical treatment and at least three months following the completion of radiotherapy, chemotherapy, and immunotherapy. Participants with a history of infectious diseases, autoimmune disorders, chronic conditions, immunodeficiency and hydrops were excluded from the study. The study population included patients with five types of solid tumors: lung, colorectal, breast, gastric, and thyroid cancers, as well as other malignancies. Each cancer type was categorized as either primary or metastatic, based on the presence or absence of distant metastasis. The cohort's average age was 54 years, with the youngest participant being 16 and the oldest 85. The study included 240 male and 280 female patients. A total of 109 age- and sex-matched healthy volunteers were recruited for the control research. The Ethics Committee of Shanghai Mengchao Cancer Hospital approved this study, which was carried out in conformity with relevant guidelines and regulations. All subjects provided informed consent, and the study adhered to the confidentiality provisions of the patients as well as the ethical norms of the Declaration of Helsinki. Flow cytometry detected six phenotypic markers (CD3, CD4, CD8, CD14, CD16, and CD56), and an automatic blood analyzer detected absolute counts. This study focused on the differences of immune cells in PBMC between tumor patients and healthy individuals, as well as the effects of age, sex, and tumor metastasis.

### Apheresis of peripheral blood mononuclear cell

The PBMC of patients from Shanghai Mengchao Cancer Hospital Affiliated to Shanghai University were collected through a blood cell separator (Fresenius Kabi), which is mainly composed of a separation chamber, a pump, and an infusion device. Extracorporeal circulation of the whole blood was performed in totally sealed pipes that were connected to a blood cell centrifuge and then centrifuged in accordance with the manufacturer's instructions. The whole blood was divided into different layers based on component density using the gradient centrifugation approach. After detecting the corresponding layers with the interface detector, the rest of the blood was returned to the donor. When the required volume was reached in connection with collecting cycles, the equipment would be turned off. The PBMCs were purified and cryopreserved in liquid nitrogen, which might be used in immunotherapy as necessary, such as preparation for CAR-T therapy.

### Flow cytometry assays for immunophenotyping

Five panels with distinct monoclonal antibody cocktail were constructed to identify 6 different phenotypic markers in tumor and healthy human PBMC cells (**Table 1**). OptiLyse C Lysing Solution (Beckman Coulter, USA) was used to lyse red blood cells in the mixture, which was then rinsed twice with phosphate-buffered saline (PBS). Following that, in each panel, 100 μL blood was combined with the appropriate antibody cocktail and incubated for 30 minutes in the dark at 2-8°C. The leftover nucleated cells were resuspended in 300 μL PBS and examined using CytoFLEX flow cytometry (Navios, Beckman Coulter, USA), and cell subset percentages were computed using Kaluza analysis Software.

### Assays for absolute counting

Absolute counting of PBMC samples was performed using a Sysmex Corporation XN-550 automatic hematological analyzer and XS-1000i analytical software. During sample analysis, 500 μL of samples were mixed for 10 seconds using a Vortex Mixer before being placed beneath the sample aspirating needle. The sample aspirating needle was put into the sample tube's bottom, and the start switch was pressed. The analysis results were shown and outputted after aspirating.

### Statistical analyses

SPSS statistical software version 24.0 (IBM Corporation, Armonk, NY, USA) was used for all data processing. Statistical significance was considered as a P value <0.05. The Mann-Whitney U-test model and the independent samples t-test were used to examine immune profile differences between cancer patients and healthy people. To determine data distribution, the Shapiro-Wilk test was used. Descriptive statistics such as means, medians, and standard deviations (SD) were applied. Graph Pad Prism version 9.4.1 software was used for graphing.

## Results

### Study population

A total of 520 cancer patients and 109 healthy volunteers were included in the study. **Table 2** provides a summary of the demographic characteristics of the participants. The patient population included 144, 93, 68, 45, 42, and 128 cases with lung, colorectal, breast, gastric, thyroid and other cancers, respectively. Healthy volunteers (48 men and 61 women), ranging in age from 20 to 71 years (median age: 48 years), were enrolled in the study. Ages were separated into three groups based on World Health Organization age recommendations (young, 0-44 years old; middle-aged, 45-59 years old; and elderly, 59 years and older).

### Flow cytometry assays of PBMC samples

**Figure 1:**
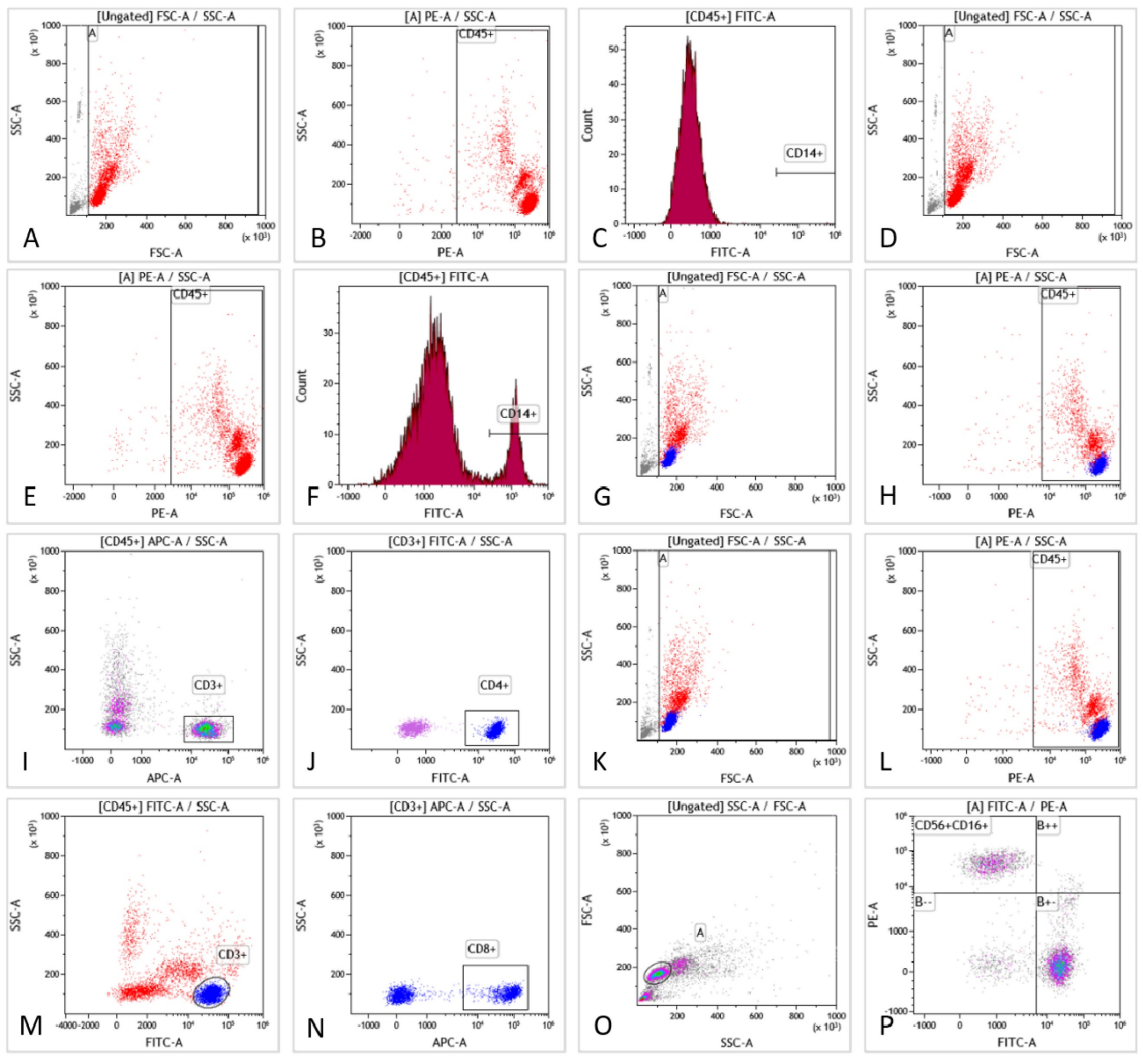
The scheme of flow cytometry detection. **Fig. 1A-C** are blank controls. Gating strategies of monocytes are presented in **Fig. 1D-F**, including forward/side scattering according to the size and granularity of the target cells (**Fig. 1D**). Leukocytes were gated according to physical characteristics (**Fig. 1E**). Monocytes were identified by CD14 staining (**Fig. 1F**). Moreover, the percentage of CD14+ monocytes was obtained. Gating strategies for T and T-helper subsets are presented in **Fig. 1G-J**, including forward/side scattering according to its size and particle size (**Fig. 1G**). Leukocytes were gated by physical characteristics and T cells were gated from CD45+ (**Fig. 1H**), which could be identified by CD3 expression (**Fig. 1I**). Additionally, the proportion of CD3+T cells was obtained. Helper T-cell subsets could be identified by CD4 staining (**Fig. 1J**). The proportion of CD3+CD4+T cells was obtained. A gating strategy for killer T cells can be seen in **Fig. 1K-N**. Gating was performed according to the size and granularity of the positive/side scattering (**Fig. 1K**). Leukocytes were gated by physical characteristics (**Fig. 1L**) and CD45+ could be identified by CD3 expression (**Fig. 1M**). Killer T cell subsets could be detected by CD8 staining (**Fig. 1N**) to obtain the proportion of CD3+CD8+T cells. NK-cell gating strategies are presented in **Fig. 1O-P**. Gating was performed according to the size and granularity of the positive/side scattering (**Fig. 1O**). NK cells were identified by CD56/CD16 expression and CD3 negative expression (**Fig. 1P**). The proportion of CD3-CD16+CD56+NK cells was obtained.

Six phenotypic markers were examined (CD3, CD4, CD8, CD14, CD16, and CD56) (**Fig. 1**). Tube 1 functioned as a blank control for tube 2. The CD14 molecule was used to identify monocytes in tube 2. Tube 3 was used to detect lymphocyte (CD3+) and T lymphocyte (CD4+) subsets. Tube 4 was employed to detect lymphocyte subsets (CD3+) and T lymphocyte subsets (CD8+). Tube 5 was used to separate the NK cell subset (CD16+CD56+) from the lymphocytes. A schematic diagram of the flow cytometry procedure is shown in Fig. 1A-P.

### Analysis of the difference of peripheral immune cell subtypes proportion

**Figure 2:**
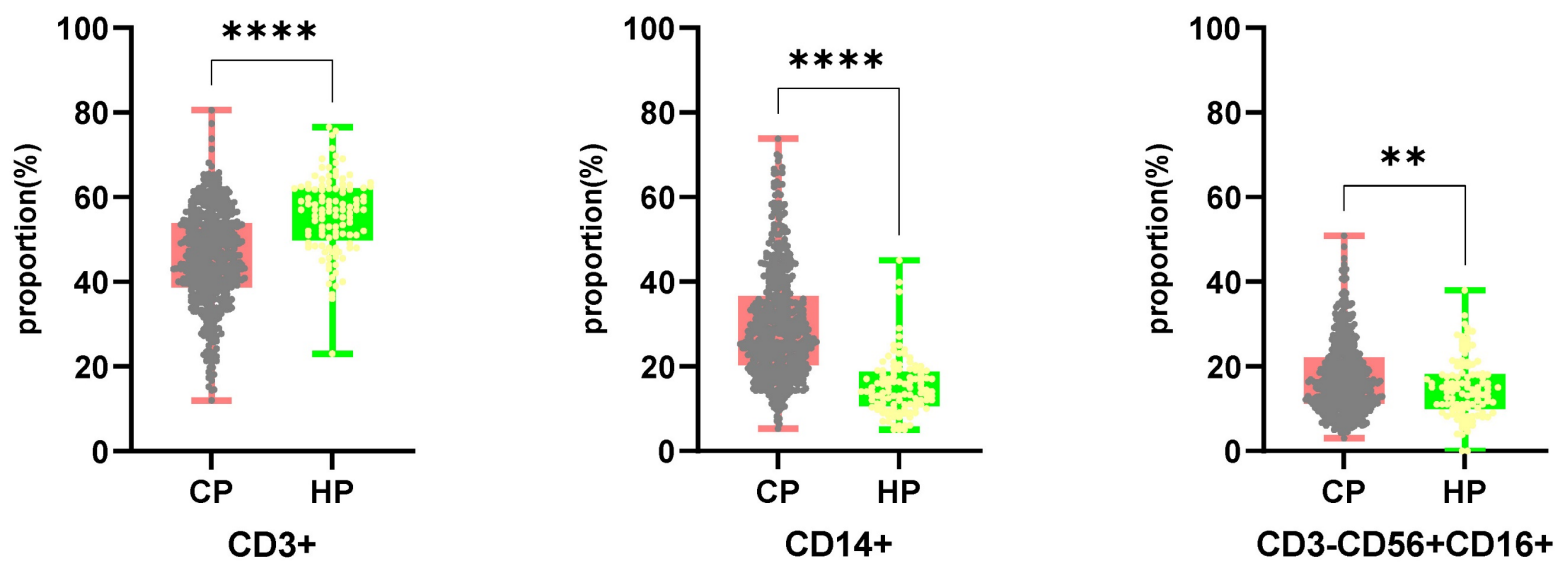
Comparing the proportion of CD3+, CD14+, CD3-CD56+CD16+ cells in PBMCs from cancer patients and healthy individuals. CP: cancer patients. HP: healthy people.

Flow cytometry was used to determine the number of T cells (including CD4 and CD8 subsets), NK cells, and monocytes in PBMC samples from 520 cancer patients and 109 healthy people. **Fig. 2** depicts the results. Cancer patients had a lower proportion of CD3+ cells than healthy individuals (45.54%/55.83%, P <0.0001), but a higher proportion of CD14+/CD3-CD56+CD16+ cells (30.04%/15.17%, P <0.0001 versus 17.45%/14.93%, P =0.0037). The ratio of CD4+/CD8+T cells did not differ significantly between cancer patients and healthy controls.

### Single factors associated with differences in the proportions of peripheral immune cell subtypes

**Figure 3:**
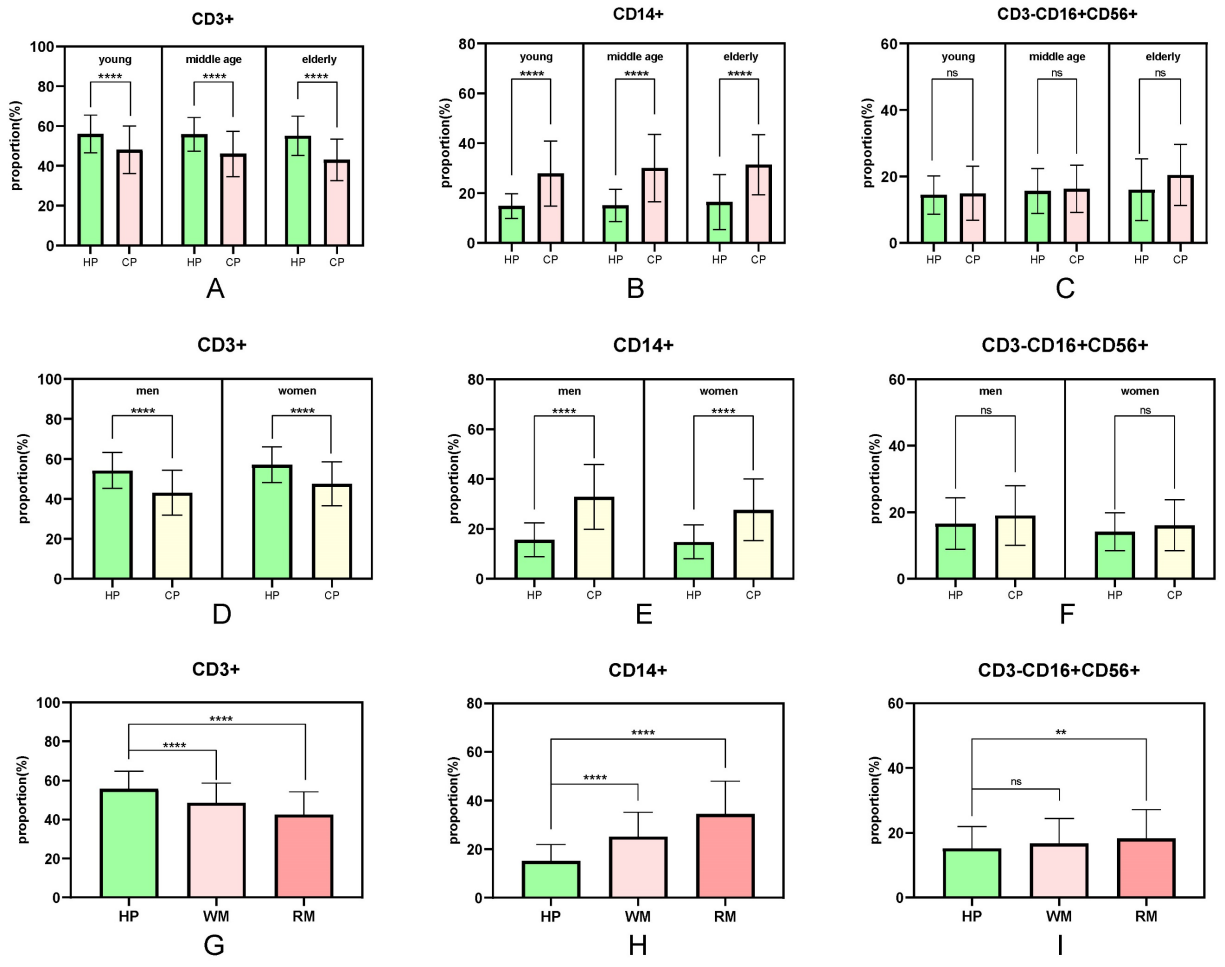
The influence of age, sex, and tumor metastasis on the difference percentage of CD3+/CD14+/CD3-CD56+CD16+ cells. WM: without metastasis; RM: remote metastasis.

The number of peripheral immune cell subsets differed between healthy people and cancer patients (**Fig. 3**). When compared to healthy people of the same age, the proportion of peripheral immune T cells in tumor patients in the young, middle-aged, and elderly groups showed a significant trend of decline (P <0.0001), and the proportion of T cells in the tumor group gradually decreased with age (young 48.12%, middle-aged 46.09%, and elderly group 43.16%) (**Fig. 3A**). Similarly, the proportion of peripheral immune monocytes in the young, middle-aged, and elderly groups increased significantly when compared to healthy adults of the same age (P <0.0001 for all three groups). The proportion of monocytes in the cancer group increased steadily with age (youth group 27.87%, middle-aged group 30.10%, and elderly group 31.41%) (**Fig. 3B**). There was no significant difference in the proportion of peripheral immunological NK cells between the young, middle-aged and elderly groups (P =0.7110, P =0.5626, and P =0.0723, respectively) (**Fig. 3C**).

The fraction of peripheral immune cell subsets that differed between healthy people and cancer patients were also associated to sex (**Fig. 3D-F**). The proportion of peripheral immune T cells in male and female cancer patients decreased significantly (P <0.0001) when compared to healthy individuals of the same sex, with male patients experiencing a greater decrease than female patients (healthy individuals 55.83%/female patients 47.61%/male patients 43.12%) (**Fig. 3D**). Similarly, the proportion of peripheral immune monocytes was significantly higher in male and female cancer patients than in healthy people of the same sex (P <0.0001), with male patients increasing more than female patients (healthy individuals 15.17%/female patients 27.62%/male patients 32.85%) (**Fig. 3E**). There was no significant difference in the proportion of peripheral immunological NK cells both in the male groups and female groups (**Fig. 3F**).

The proportion of peripheral immune cell subsets that differ between healthy people and cancer patients is also connected to the degree of tumor malignancy (**Fig. 3G-I**). The drop trend of CD3+ T cells in the non-metastatic and the metastatic groups was remarkably different (P <0.0001) when compared to the healthy group, and the downward tendency was intensified (healthy group 55.83%, non-metastatic group 48.63%, metastatic group 42.59%) (**Fig. 3G**). Similarly, the percentage of CD14+ monocytes increased significantly (P <0.0001), and the growing trend was accelerated (healthy group 15.17%, non-metastatic group 25.14%, metastatic group 34.51%) (**Fig. 3H**). The proportion of CD3-CD16+CD56+ peripheral immunity NK cells in metastatic groups increased significantly (P =0.0017) when compared to healthy individuals and there was no significant difference in non-metastatic groups (P =0.0821) (**Fig. 3I**).

### Multiple factors associated with differences in the proportions of peripheral immune cell subtypes

Some P values did not conform to the overall trend when assessing the influence of a single factor on the difference in the proportion of cells between all healthy participants and patients with different tumor types. The influence of several factors on the differential in the number of peripheral immune cells in healthy individuals and patients with different tumor types was investigated in this section. In lung cancer patients, the young/female/non-metastatic combination had the best immune status, while the old/male/metastatic combination had the worst one.

**Figure 4:**
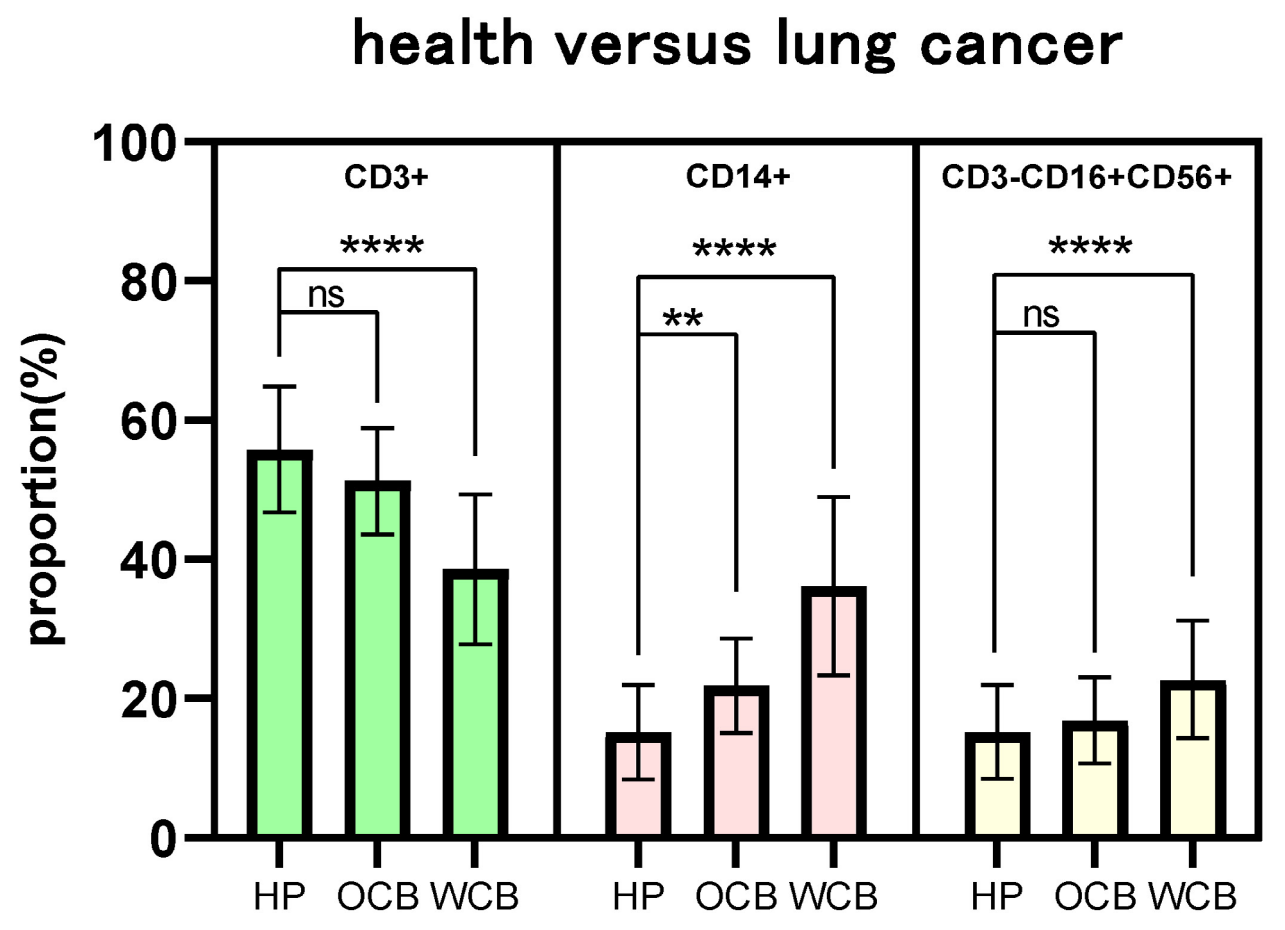
The influence of multi-factor combination on the difference of proportion of cell percentage. OCB: optimal combination, WCB: worst combination.

The proportions of CD3+ T cells, CD14+ monocytes, and CD3-CD16+CD56+ NK cells in the best immune status groups were closer to healthy groups than those in the worst groups (**Fig. 4**). There was no significant change in the proportion of CD3+ T cells and CD3-CD16+CD56+ NK cells (P =0.1427, P= 0.4708) in the best immune status groups of lung cancer compared with the healthy groups, and the p-value of the proportion of CD14+ monocytes was 0.0053. As a comparison, the proportions of CD3+T cells, CD14+ monocytes, and CD3-CD16+CD56+ NK cells in the lung cancer patients with the poorest immunological function were significantly different from those in the healthy control group (P <0.0001). It demonstrated that aging, sex, and tumor metastasis all contribute to the considerable difference in peripheral immune cell proportions between tumor patients and healthy people.

### Absolute counting of peripheral immune cells in healthy people and cancer patients

**Figure 5:**
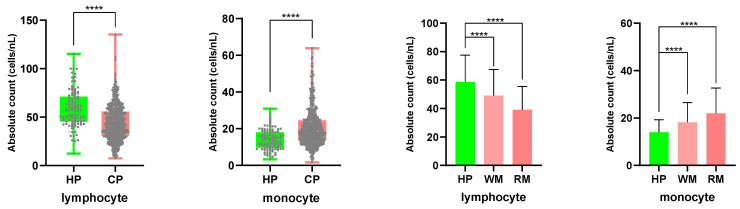
Comparing the absolute counts of lymphocyte and monocyte cells in PBMCs from cancer patients and healthy participants.

PBMC samples from 520 cancer patients and 109 healthy people were counted using an automatic hematology analyzer, and the counting data was examined (**Fig. 5**). In cancer patients, the absolute counting of lymphocytes declined (healthy groups 58.61 cells/nL; tumor patients 43.96 cells/nL), while the absolute counting of monocytes increased (healthy groups 13.96 cells/nL; tumor patients 20.25 cells/nL). The results were statistically significant (P<0.0001). The disparities in absolute lymphocyte and monocyte counts between patients with tumor metastasis and healthy individuals were larger than those between patients with non-metastasis and healthy individuals. The mean lymphocyte absolute counting was 49.15 cells/nL in the non-metastasis group and 39.15 cells/nL in the metastatic one, whereas the mean monocyte absolute counting was 18.15 cells/nL and 22.00 cells/nL, respectively.

## Discussion

Multiple immunotherapeutic-related biomarkers associated with tumor tissue or the tumor microenvironment have been used in clinical research. However, although these biomarkers were considered to be effective predictors of immunotherapy outcomes, their predictive efficacy may be conditional, with low repeatability, sensitivity, and specificity 21, 22. The proportion of lymphocyte subsets reflects lymphocyte development and differentiation ability, whereas absolute count reflects lymphocyte proliferation ability. Evidence shows that both tumor recurrence and metastasis are directly associated with the deterioration of immune function and tumor immune escape 23. It is thus important to evaluate the immune status of cancer patients by analysis of the immune cells in the peripheral blood.

We examined the percentages of CD3+, CD3+CD4+, CD3+CD8+, CD14+, and CD3-CD16+CD56+ cells in PBMC samples from 520 cancer patients and 109 healthy individuals, as well as the factors that affected them. When compared to healthy individuals, the proportion of CD3+ cells in tumor patients' PBMC significantly dropped (P <0.0001), but the proportion of CD14+ cells and CD3-CD16+CD56+ cells remarkably increased (P <0.0001, P =0.0037) (**Fig. 2**). Interestingly, we found that the proportion and absolute counting of CD3+, CD14+, and CD3-CD16+CD56+ cells in patients with thyroid cancer are the closest to those in healthy groups, whereas patients with liver cancer and cholangiocarcinoma are quite different from those in healthy groups, which may indicate that patients with thyroid cancer have relatively light immunosuppression, low metastatic ability, and are treatable.

The results of the univariate analysis showed that the differences in the proportion of peripheral immune cells were affected by age, sex, and tumor metastasis. With increasing age, the proportion of CD3+ cells decreased overall (**Fig. 3A**), and the proportion of CD14+ (**Fig. 3B**) and CD3-CD16+CD56+ cells (**Fig. 3C**) increased, which was consistent with the influence of tumor on the proportion of immune cells compared with healthy individuals. This trend was also observed in the male group compared to the female one, and in the metastatic patient group compared to the non-metastatic one. Men's and women's immune systems vary in composition and signaling pathways. Sex influences how male and female immune systems control and respond to aberrant stimulation or tumor formation 24, 25. Our investigation confirmed the difference between men and women in proportion of immune cells between cancer patients and healthy people (**Fig. 3D-F**). The proportion and amount of peripheral blood lymphocytes in patients with metastatic tumors were lower than in individuals with non-metastatic tumors (**Fig. 3G-I, Fig. 5**). When tumor patients were further grouped according to tumor type, similar significance was observed. A multi-factor comprehensive analysis revealed that aging, sex, and tumor metastasis could aggravate the difference in the proportion of peripheral immune cells (**Fig. 4**).

There is no question that tumors can harm the body's immune system by impairing the function of various immune cell types. The results of many studies on the absolute numbers and proportions of immune cells are consistent with our findings that the absolute lymphocyte counts of patients decreased while the proportions of NK cells and monocytes increased 20, 26-29. T lymphocyte subsets are drastically reduced in cancer patients, indicating that the body's cellular immune activity is repressed and the ability to recognize and kill mutant cells is diminished. Moderate proportions and ratios of T cells in PBMC indicate healthy immunity and may contribute to a favorable prognosis 30, 31. Increased percentage of NK cells in colorectal cancer patients and monocytes in melanoma patients were discovered compared to healthy donors 19, 32. Although monocytes and NK cells play an important role in the immune destruction of tumor cells, an overly non-reasonable proportion may result in immunosuppression, which is not conducive to cytotoxicity against tumor cells.

Nevertheless, pathological diagnosis currently remains the gold standard for tumor diagnosis. However, compared with the inspection of tumor tissue, peripheral blood sampling is simple, less traumatic, representative, and can be repeatedly performed 33. Investigations into alterations in the numbers and proportions of PBMC cells between tumor patients and healthy individuals have already started 34-36. Studies have shown that the presence of CD8+ tumor-infiltrating lymphocytes is associated with favorable patient prognosis while the presence of immunosuppressive regulatory T cells (Tregs) and macrophages is adversely correlated with survival outcomes 37. Circulating NK cells and monocytes in the peripheral blood can also be used as indicators of the likelihood of progression-free survival and overall survival 32, 38. The limitation of the present study is that the morphological assessment of cells was relatively limited, with no additional investigation of specific cell phenotypes. Furthermore, the previous treatments of the patients were not investigated, and the impact of different treatments on the immune system should be considered in further studies. We intend to investigate this issue to determine the effects of the different conditions of cancer patients on their prognosis as evaluated by PMBCs. Furthermore, considering today's advanced cryopreservation technology, keeping PBMC at a young age or at an early stage of the disease can considerably improve the efficiency of tumor immunotherapy, which has important implications for future cancer treatment.

We evaluated the proportion and absolute count of peripheral immune cells in healthy individuals and tumor patients using a large sample size and multiple tumor types. Our findings revealed that the average proportion of T cells in the PBMC population showed a significant downward trend while the proportion of monocytes and NK cells showing a significant upward trend in tumor patients compared to healthy volunteers. Three factors aging, sex, and tumor metastasis, all had a remarkable impact on the difference in peripheral immune cell proportions. Aging, being male, and tumor metastasis exacerbated the difference in the number of CD3+ cells, CD14+ cells, and CD3-CD16+CD56+ cells between cancer patients and healthy participants.

## Figures and Tables

**Table 1:** Antibody composition of five panels for differentiating cell phenotype

Fluorochrome	PE	FITC	APC
Panel 1	CD45	/	/
Panel 2	CD45	FITC Anti-human CD14	/
Panel 3	CD45	CD4	CD3
Panel 4	CD45	CD3	CD8
Panel 5	CD16+CD56	CD3	/

**Table 2:** Characteristics of study participants

Category	LC	CC	BC	GC	TC	OC	HP
N	144	93	68	45	42	128	109
Sex							
male	80	60	0	27	15	58	48
female	64	33	68	18	27	70	61
Age							
≤45	27	21	23	9	15	22	52
45-59	50	31	35	20	23	62	46
≥60	67	41	10	16	4	44	11
Tumor metastasis							
WM	80	19	39	16	33	53	/
RM	59	73	29	28	8	74	/
UN	5	1	0	1	1	1	/

LC: lung cancer; CC: colorectal cancer; BC: breast cancer; GC: gastric cancer; TC: thyroid cancer; OC: other cancers; HP: healthy people; WM: without metastasis; RM: remote metastasis; UN: uncertain.

## Abbreviations

PBMC: peripheral blood mononuclear cell
PBS: phosphate-buffered saline
LC: lung cancer
CC: colorectal cancer
BC: breast cancer
GC: gastric cancer
TC: thyroid cancer
OC: other cancers
HP: healthy people
CP: cancer patients
WM: without metastasis
RM: remote metastasis
UN: uncertain
OCB: optimal combination, WCB: worst combination
